# Supplementary material for: Early Succession of Community Structures and Biotic Interactions of Gut Microbes in Eriocheir sinensis Megalopa after Desalination
Source: Microorganisms. 2024 Mar 11;12(3):560. doi: 10.3390/microorganisms12030560 (PMC10974759; doi:10.3390/microorganisms12030560)
Supplement: Supplementary file 1 [file microorganisms-12-00560-s001.zip › microorganisms-2888986-supplementary.pdf]

Supplementary Material

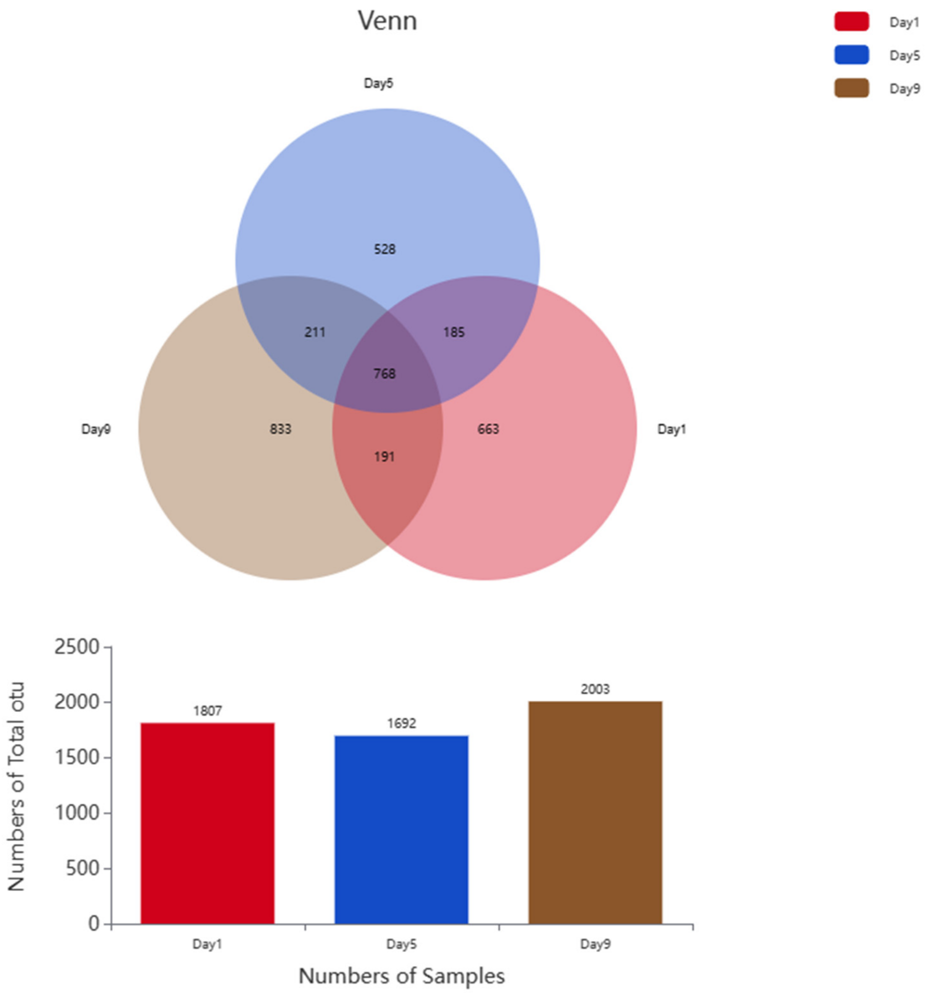

Supplementary Figure S1. Venn diagram showing the number of shared and unique OTUs in the Day1, Day5 and Day9 groups.

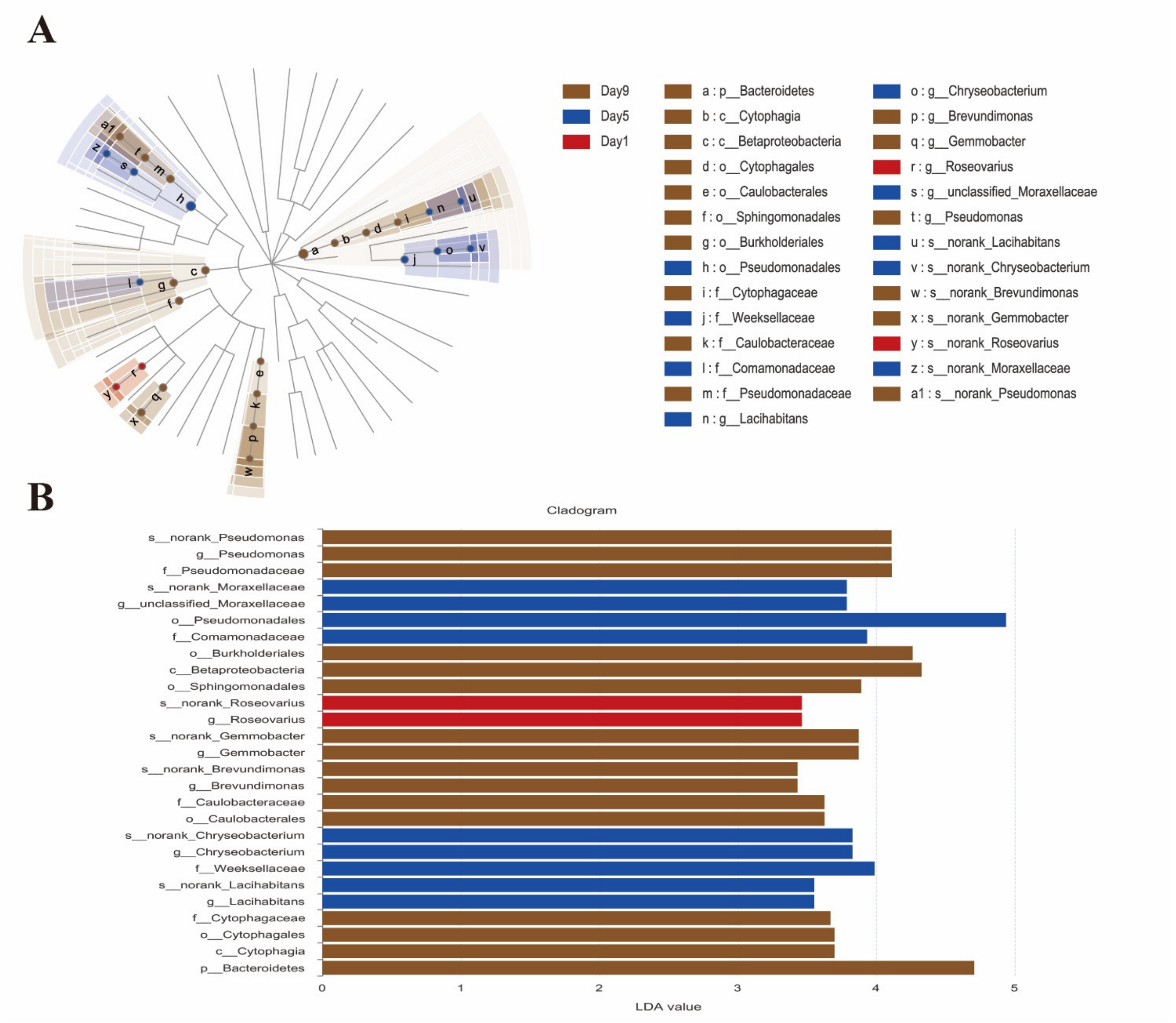

Supplementary Figure S2 Intergroup variation in the relative abundance of intestinal microbial communities. (A) Cladogram from Lefse. (B) LDA score from the phylum to species level.

TABLE S1. Effects of sampling sites and time on the gut microbial community compositions of Genus level. Here, changes in the 10 dominant genera were determined using the linear mixed-effects model of "y ~ Time × Site + (1|Rep)", where the "Rep" represent the replication groups. Type III analysis with Satterthwaite's method was used to determine if differences across sampling time and sites were significant. We show the results of F and significant P values in the table ($^{*}0.01 < P \leq 0.05$; $^{**}0.001 < P \leq 0.01$; $^{***}P \leq 0.001$).

Genus	Site	Time	Site * Time
Acinetobacter	1.12	0.25	0.54
Bosea	**3.13***	0.40	**2.86***
Jatrophihabitans	0.08	**3.21***	0.25
Mycobacterium	0.07	**5.14****	0.57
Nakamurella	0.13	**5.29****	1.39
Phycicoccus	0.14	**2.94***	0.38
Pseudomonas	1.34	**6.06****	2.45
Rhizobium	2.55	1.78	**2.73***
Unassigned	**12.43*****	**2.83***	**4.83***
Others	1.18	**3.97****	**2.37***
